# Interactions among Interleukin‐1β, Amyloid‐β, and Tau drive synaptic deficits in a mouse model of sporadic Alzheimer's disease induced by HSV‐1 infection

**DOI:** 10.1002/alz70855_102964

**Published:** 2025-12-23

**Authors:** Domenica Donatella Li Puma, Roberto Piacentini, Giammarco Boni, Gulia Puliatti, Marco Rinaudo, Giovanna De Chiara, Anna Teresa Palamara, Claudio Grassi

**Affiliations:** ^1^ Università Cattolica del Sacro Cuore, Rome, Rome, Italy; ^2^ Fondazione Policlinico Universitario A. Gemelli IRCCS, Rome, Italy; ^3^ Università Cattolica del Sacro Cuore, Rome, Italy; ^4^ Institute of Translational Pharmacology, CNR, Rome, Rome, Italy; ^5^ University of Rome Sapienza, Rome, Rome, Italy; ^6^ Istituto Superiore di Sanità, Rome, Italy

## Abstract

**Background:**

We recently demonstrated that reactivation of Herpes Simplex Virus type 1 (HSV‐1) in the mouse brain, triggered by thermal stress (TS), induces an Alzheimer's disease (AD)‐like phenotype characterized by synaptic and memory deficits, elevated interleukin‐1β (IL‐1β) levels, and the accumulation of amyloid‐β (Aβ) and phosphorylated Tau (pTau) proteins. Treatment with the IL‐1 receptor blocker anakinra reversed the structural and functional markers of neurodegeneration observed after 2TS. Here, we investigated the interplay among IL‐1β, Aβ, and Tau in driving synaptic dysfunction in infected mice.

**Method:**

Following HSV‐1 infection and 2TS‐induced viral reactivations, molecular, electrophysiological, and behavioral analyses were conducted on C57BL/6 wild‐type (WT) and transgenic mice deficient in either Aβ (APP^‐/‐^) or pTau (Tau^‐/‐^) production.

**Result:**

After 2TS, HSV‐1‐infected Tau^‐/‐^ and APP^‐/‐^ mice exhibited higher IL‐1β mRNA levels than mock‐infected mice (1.6‐ and 1.4‐fold increases, respectively, *p* <0.05). However, IL‐1β levels were significantly lower than those observed in HSV‐1‐infected WT mice (‐32% and ‐43%, respectively, *p* <0.05). The decreased concentration of IL‐1β in HSV‐1‐infected transgenic mice correlated with reduced microglial activation. In particular, the upregulation of the M1 microglial marker CD86 was significantly lower in HSV‐1‐infected Tau^‐/‐^ and APP^‐/‐^ mice (+63% and +64% vs mock‐infected mice, respectively) compared to the much greater increase observed in WT mice (+197%).

Accordingly, transgenic mice showed milder synaptic deficits than WT mice. Specifically, hippocampal long‐term potentiation (LTP) in HSV‐1‐infected vs mock‐infected mice was: 73.7±6.7% vs 98.6±10.1% in Tau^‐/‐^, 69.5±5.6% vs 94.6±6.4% in APP^‐/‐^, and 47.3±6.4% vs 98.0±12.4% in WT mice. HSV‐1‐infected mice also exhibited memory impairments, as assessed by the novel object recognition test. Specifically, the preference index was: 50.7±1.3% vs 62.0±1.6% in Tau^‐/‐^, 55.7±2.2% vs 62.5±2.1% in APP^‐/‐^, and 51.6±2.1% vs 65.2±1.6% in WT mice (*p* <0.05 for all groups).

**Conclusion:**

Our findings suggest that IL‐1β‐mediated neuroinflammation plays a pivotal role in driving synaptic dysfunction during the early stages of this mouse model of AD. The lower IL‐1β levels and reduced synaptic deficits observed in mice lacking either Aβ or pTau indicate that these key AD hallmarks contribute to IL‐1β accumulation, thereby triggering a vicious circle in the pathophysiology of synaptic dysfunction in AD.

